# Supplementary material for: SyNDI: synchronous network data integration framework
Source: BMC Bioinformatics. 2018 Nov 6;19:403. doi: 10.1186/s12859-018-2426-5 (PMC6219086; doi:10.1186/s12859-018-2426-5)
Supplement: Supplementary file 5 — Files of the example presented in Additional file 4. This additional file is a zip package that contains all files used in Additional file 4. Detailed descriptions of each of these files are presented in a README file included in this zip package. (GZ 30411 kb) [file 12859_2018_2426_MOESM5_ESM.gz › SyNDITools/Meme2Fimo/meme_4.9.0_4.tar.gz!/meme_4.9.0/doc/mhmm-output.html]

MHMM Output


# MHMM Output

**The Meta-MEME model file contains the following
information:**

- The Meta-MEME version number and release date.
- The program name, file contents and date the file was
  created.
- A link to the Meta-MEME home page.
- A reference to cite if you publish results from Meta-MEME.
- Some global information about the Meta-MEME model.
- A list of the states in the Meta-MEME model.
- The matrix of transition probabilities between states of the
  model.

  
  

**HMM Global Information**

The list of states in the Meta-MEME model begins with eight lines
that specify the global characteristics of the model. These lines
tell  
  

- whether the model has a linear or completely connected topology
  (i.e., whether the model allows for repetition and shuffling of
  motifs),
- the total number of states in the model,
- the total number of spacers (i.e., inter-motif regions) in the
  model,
- the number of states used to represent each spacer,
- the number of letters in the alphabet (4 for DNA and 20 for
  amino acids),
- the alphabet,
- the background frequencies used as emission probabilities in
  the spacer states, and
- the name of the MEME output file that was used to generate this
  Meta-MEME model.

  
  

**HMM States**

Following the global characteristics is a list of state
descriptions. Each state description consists of six lines. These
lines tell

- the index of the state (numbered from 0),
- the type of state (i.e, whether it is a start or end state, a
  spacer state, or the beginning, middle or end of a motif),
- the index of the motif within which this state appears (This
  value is arbitrarily set to -1 for non-motif states.),
- the position of the state within the motif or spacer region,
  and
- the letter that is printed above this state when the MEME motif
  ID is printed centered with respect to the motif,
- the list of probabilities that this state will emit each of the
  letters in the alphabet.

  
  

The final item (the list of emission probabilities) is omitted
from the start and end states, which do not emit letters.

  
  

**Transition probability matrix**

The Meta-MEME model file ends with a square matrix containing n
rows and n columns, where n is the number of states in the model.
The entry in row i, column j of this matrix is the probability of
transitioning from state i to state j in the model. Consequently,
each row in the matrix sums to 1.0.
